# Multimodal deep learning models for the prediction of pathologic response to neoadjuvant chemotherapy in breast cancer

**DOI:** 10.1038/s41598-021-98408-8

**Published:** 2021-09-22

**Authors:** Sunghoon Joo, Eun Sook Ko, Soonhwan Kwon, Eunjoo Jeon, Hyungsik Jung, Ji-Yeon Kim, Myung Jin Chung, Young-Hyuck Im

**Affiliations:** 1 Technology Research, Samsung SDS, Seoul, 06765 Republic of Korea; 2 Department of Radiology, Samsung Medical Center, Sungkyunkwan University School of Medicine, 81 Irwon-ro, Gangnam-gu, Seoul, 06351 Republic of Korea; 3 Division of Hematology/Oncology, Department of Medicine, Samsung Medical Center, Sungkyunkwan University School of Medicine, 81 Irwon-ro, Gangnam-gu, Seoul, 06351 Republic of Korea; 4 Present Address: VUNO Inc., Seoul, 06536 Republic of Korea

**Keywords:** Breast cancer, Computational science, Information technology

## Abstract

The achievement of the pathologic complete response (pCR) has been considered a metric for the success of neoadjuvant chemotherapy (NAC) and a powerful surrogate indicator of the risk of recurrence and long-term survival. This study aimed to develop a multimodal deep learning model that combined clinical information and pretreatment MR images for predicting pCR to NAC in patients with breast cancer. The retrospective study cohort consisted of 536 patients with invasive breast cancer who underwent pre-operative NAC. We developed a deep learning model to fuse high-dimensional MR image features and the clinical information for the pretreatment prediction of pCR to NAC in breast cancer. The proposed deep learning model trained on all datasets as clinical information, T1-weighted subtraction images, and T2-weighted images shows better performance with area under the curve (AUC) of 0.888 as compared to the model using only clinical information (AUC = 0.827, *P* < 0.05). Our results demonstrate that the multimodal fusion approach using deep learning with both clinical information and MR images achieve higher prediction performance compared to the deep learning model without the fusion approach. Deep learning could integrate pretreatment MR images with clinical information to improve pCR prediction performance.

## Introduction

Neoadjuvant chemotherapy (NAC) is increasingly used in the management of early and/or locally advanced breast cancer^1^. NAC has the following potential benefits for breast cancer therapy: preventing micrometastasis preoperatively to increase overall survival, downsizing tumors to make surgery feasible, and providing in vivo assessments of sensitivity to a chemotherapy^2–4^. After NAC, the achievement of the pathologic complete response (pCR) has been considered a metric for the success of NAC and a powerful surrogate indicator of the risk of recurrence and long-term survival^5,6^.

Recently, many studies have been conducted to find clinical and pathologic features to predict pCR prior to the NAC^7–9^. As a monitoring tool, breast magnetic resonance (MR) imaging has been shown to be most effective in predicting response to NAC^10^. Recently, several studies using radiomics methodology in which features were extracted in the computational method from breast MR images measured after the first cycle of NAC or even pretreatment have shown promise in predicting pCR^11–13^. However, radiomic studies have limited values because of the reproducibility issue of radiomics features, which could be affected by different radiomics software or MR acquisition parameters.

To overcome the limitations of previous studies, deep learning could be applied. The deep learning method extracts all possible features from a dataset by learning its representation with minimal human intervention^14^. In medical image analysis, convolutional neural network (CNN) has shown high performance in classification and segmentation tasks^15–18^. From breast MR images, several attempts with deep learning methods have been conducted to predict pCR to NAC in patients with breast cancer^19–21^. Recent studies have reported that the performance of the deep learning model is improved when the fusion approach that integrates features from MR images which have different views, scales, or acquisition protocols, and even clinical information was applied in diagnosing breast cancer^15,22–24^. However, previous studies were conducted with a relatively small number of patients or limited clinical information or using just a few select images not covering the entire tumor. This provokes the need of an in-depth research of the application of deep learning to fuse MR images with clinical information for predicting pCR to NAC in patients with breast cancer.

This study aimed to develop a deep learning model to fuse high-dimensional MR image features and the clinical information for the pretreatment prediction of pCR to NAC in breast cancer. We designed the architecture of deep neural network that combined ResNet-50 with 3D CNNs for MR images and fully connected (FC) layers for clinical information.

## Results

### Patients’ outcome and characteristics

Table 1 shows the general characteristics of the patients enrolled in this study. Overall, there were 133 (24.8%) and 403 (75.2%) patients with and without pCR, respectively. More than half of the patients were ER negative (*n* = 299, 55.8%), PR negative (*n* = 342, 63.8%), and HER2 negative (*n* = 359, 67.0%). Most patients were treated with AC-T regimen (*n* = 367, 68.5%).

**Table 1:** General characteristics of the study population.

	Total (*N* = 536 patients)	pCR (*N* = 133 patients)	Non-pCR (*N* = 403 patients)	*P* value
**Age (y)**				
Mean (± standard deviation)	45.22 (± 9.91)	46.13 (± 10.13)	44.92 (± 9.82)	0.222
**CA 15–3 (U/mL**)				0.024
Mean (± standard deviation)	17.52 (± 23.59)	13.53 (± 15.41)	18.84 (± 25.61)	
ER (n, %)*				
Positive	266 (49.63)	51 (19.17)	215 (80.83)	0.002
Negative	270 (50.37)	82 (30.37)	188 (69.63)	
PR (n, %)*				
Positive	209 (38.99)	31 (14.83)	178 (85.17)	< 0.001
Negative	327 (61.01)	102 (31.19)	225 (68.81)	
**HER2 (n, %)**				
Positive	177 (33.02)	65 (36.72)	112 (63.28)	< 0.001
Negative	359 (66.98)	68 (18.94)	291 (81.06)	
**Pathologic diagnosis (n, %)**				
IDC	481 (89.74)	127 (26.40)	354 (73.60)	0.012
Others	55 (10.26)	6 (10.91)	49 (89.09)	
**Ki-67 (n, %)**				
1 +	102 (19.03)	14 (13.73)	88 (86.27)	0.002
2 +	179 (33.40)	44 (24.58)	135 (75.42)	
3 +	128 (23.88)	30 (23.44)	98 (76.56)	
4 +	127 (23.69)	45 (35.43)	82 (64.57)	
**Clinical T-stage at diagnosis (n, %)**				
cT1	26 (4.85)	12 (46.15)	14 (53.85)	< 0.001
cT2	288 (53.73)	86 (29.86)	202 (70.14)	
cT3	178 (33.21)	25 (14.04)	153 (85.96)	
cT4	44 (8.21)	10 (22.73)	34 (77.27)	
**Clinical N-stage at diagnosis (n, %)**				
cN0	42 (7.84)	12 (28.57)	30 (71.43)	0.032
cN1	108 (20.15)	38 (35.19)	70 (64.81)	
cN2	230 (42.91)	50 (21.74)	180 (78.26)	
cN3	156 (29.10)	33 (21.15)	123 (78.85)	
**NAC regimen (n, %)**				
AC-T	367 (68.47)	75 (20.44)	292 (79.56)	< 0.001
ACTH	131 (24.44)	51 (38.93)	80 (61.07)	
AC-T & Platinum	15 (2.80)	4 (26.67)	11 (73.33)	
AC	23 (4.29)	3 (13.04)	20 (86.96)	

*For ER and PR, the Allred scores (0–8) are used in the actual training and validation procedures. For convenience, they are dichotomized in this table.

### Model performance for predicting pCR

**Figure 1:**
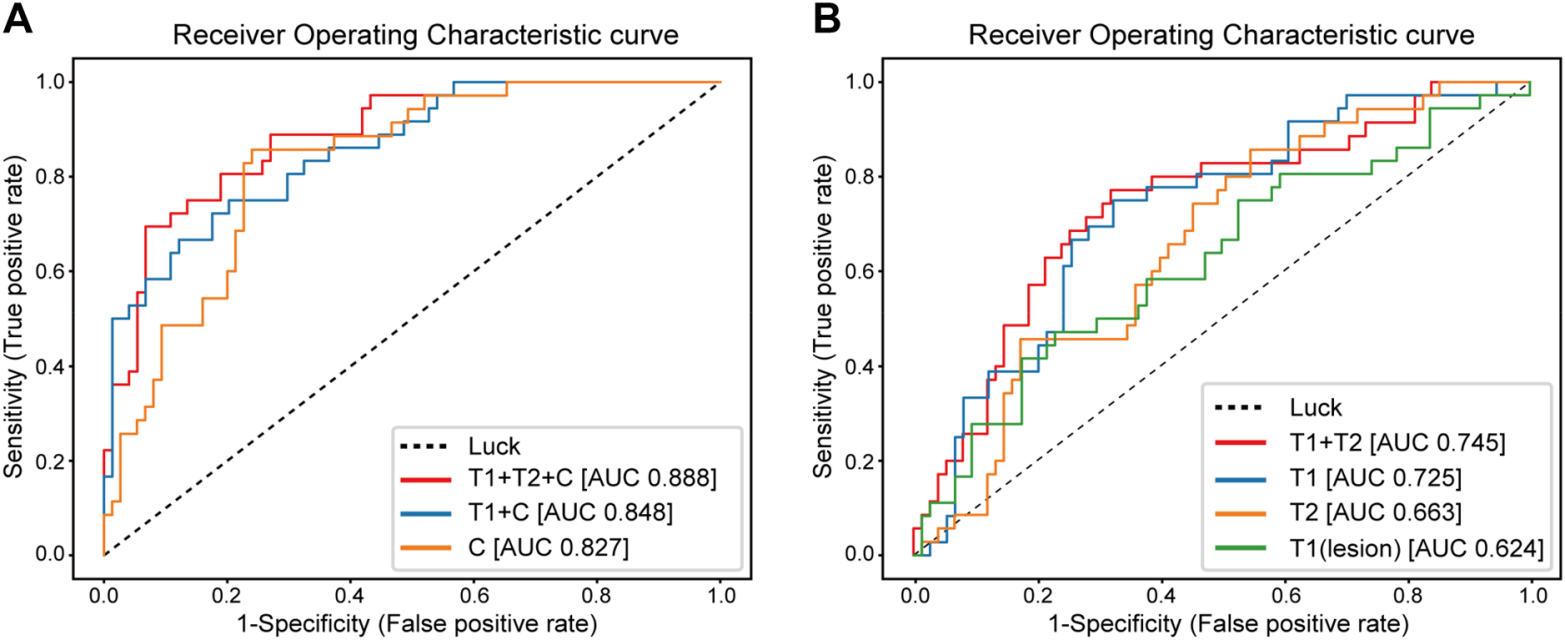
Receiver operating characteristic curves showing the AUC values of different deep learning models in the validation set. (**A**) ROC curves for the prediction of pretreatment pCR based on different deep learning models trained on clinical information and MR images or only clinical information pCR classifiers. T1 + T2 + C: subtracted-T1W images, T2W images, and clinical information. T1 + C: T1W subtraction images and clinical information. C: clinical information. (**B**) ROC curves for prediction of pretreatment pCR based on different deep learning models trained on the dataset in the combinations of MR images. T1 + T2: T1W subtraction images and T2W images. T1: T1W subtraction images. T2: T2W images. T1 (lesion): cropped image of the lesion in T1W subtraction images.

To determine the training and validation sets, the entire patient group was randomly divided into the training set (*n* = 429) and validation set (*n* = 107). There was no significant difference between the training and validation sets, except in T stages (*P* = 0.047) (Supplementary Table S1). The performances are shown in Table 2 and Fig. 1. We found that the deep learning model trained on the dataset that contained paired T1-weighted (T1W) subtraction images, T2W images, and clinical information demonstrated the highest accuracy for predicting pCR in the validation set (area under the curve [AUC] = 0.888). The clinical information-trained deep learning model achieved a validation AUC of 0.827. Comparison of AUC showed a significant difference between the two models (*P* < 0.05). We investigated the relative importance of each clinical information as input on the performance of the deep learning model. Supplementary Figure S1 shows the importance of each clinical information to the deep learning model. The type of NAC regimen and Ki-67 level showed relatively high importance compared to other features in the clinical information. The performance in predicting pCR of the model trained on the dataset with T1W subtraction images and clinical information (AUC = 0.848) was higher than that of the model trained on only clinical information. According to the ROC curve for these three deep learning models in Fig. 1A, the performance of the deep learning models improved with the type of data used for training. Confusion matrices include information about actual and predicted classifications for models (Supplementary Figure S2).

**Table 2:** Performance of seven deep learning models in a validation dataset in predicting pCR.

Input modality	AUC (S.E.)	Accuracy	Sensitivity	Specificity	PPV	NPV
Clinical information	0.827 (0.027)	0.785	0.848	0.757	0.609	0.918
T1W subtraction image	0.725 (0.031)	0.718	0.314	0.907	0.601	0.748
T2W image	0.663 (0.033)	0.709	0.457	0.824	0.537	0.773
Cropped T1W subtraction image for lesion (56 × 56 × 12)	0.624^#^ (0.033)	0.700	0.429	0.813	0.506	0.762
T1W subtraction + T2W images	0.745* (0.031)	0.736	0.486	0.853	0.596	0.788
T1W subtraction image + clinical information	0.848 (0.025)	0.822	0.485	0.973	0.889	0.809
T1W subtraction image + T2W image + clinical information	0.888* (0.022)	0.850	0.667	0.932	0.814	0.863

* Statistically significant difference (*p* < 0.05) compared to Clinical information. ^#^ Statistically significant difference (*P* < 0.05) compared to T1W subtraction image.

Figure 1B shows that ROC curves for the deep learning models in the validation set according to different scenarios. The deep learning model trained on T1W subtraction images and that on T2W images achieved an AUC of 0.725 and 0.663, respectively. When using cropped T1W subtraction images for the lesion as the dataset, the performance reduced with an AUC of 0.624. Compared with the deep learning model trained on uncropped T1W subtraction images, there was significant difference in AUC (*P* < 0.05). Compared to all deep learning models trained with single MR imaging sequence, the model that integrated T1W subtraction and T2W MR images had a higher prediction performance (AUC = 0.745).

## Discussion

In this study, we present a deep learning model that is capable of predicting pCR to NAC in patients with breast cancer based on pretreatment MR images. We hypothesized that the use of both clinical information and deep features extracted from breast MR images could improve performance in predicting pCR rather than using only clinical information or MR images. Our experimental results show that the multimodal fusion approach that combined clinical information, T1W subtraction images, and T2W images is the best method for pretreatment pCR prediction (AUC = 0.888). Considering that the performance improvement in the deep learning model as increase in number of dataset, it is relevant to previous findings that multiparametric MR images can reflect various information about the tumor^25^. Although our results showed that the clinical information only model had a lower performance than the MRI-clinical information fused model, the performance of clinical variable model could be improved if more dedicated variables are available, considering that the scalable nature of clinical variables unlike radiomic features extracted from images.

Our study has three notable strengths compared with earlier work on predicting pCR from breast MR images using the deep learning method. First, we included 3D-bilateral whole MR images covering the axilla and chest wall to include information outside the lesion. To the best of our knowledge, this is the first application of the 3D-CNN model to extract the features from the 3D-bilateral whole MR images. Further, we confirmed that the entire image-trained model had better performance than cropped MR image-trained model adopted in previous studies. It may be due to the inclusion of invisible to human eyes or missed abnormal findings in the axilla or other organs identified in the entire image. Second, we did not need to use manual or automatic segmentation for tumor area extraction. Additionally, there was no constraint on inclusion of patients with multiple masses or non-mass enhancement type tumors in the dataset. Therefore, our method is less labor intensive and includes wider implication. Lastly, we included 536 patients who satisfied the inclusion criteria for our cohort. It is the largest cohort in the development of the pCR prediction model with breast MR images. Regarding the impact of sample size on the accuracy and reliability, Cho et al. studied the scale of the training dataset to achieve high performance of the medical image deep learning system^26^. They trained CNN to classify axial Computed Tomography (CT) images into six anatomical classes (brain, neck, shoulder, chest, abdomen, pelvis). They present the trend that increasing the training dataset size increases the performance of the deep learning network, while the accuracy did not increase significantly from training size 100–200. At 200 patients in the training set, the average of classification accuracy for six anatomical classes was 95.67%, especially for neck and chest CT images, over 99%. We convince that our results from 536 patients are robust enough.

However, our study still has limitations. First, although it has been confirmed that important information has been extracted from the MR image to improve pCR prediction performance, it is difficult to understand how output features were extracted from deep learning model effects to classify pCR. This has been frequently included as a problem when applying deep learning methods to clinical settings^27^. Recently, there are several attention techniques using the backward pass or response of a feedforward propagation on the deep learning algorithm, such as Grad-CAM and attention branch network^28,29^, to provide explanations for the prediction using the deep learning model. However, in our study, it was difficult to apply visual explanation on MR images to interpret the decision-making of deep learning models because of the complicated model architectures for multimodal fusion and feature extraction from 3D MR images. Future studies will need to apply methods that reveal not only the model’s prediction of the pCR but also its basis. Second, this is a retrospective single-center study. For this reason, we have MR images from only two types of MR scanners purchased from a single vendor (Philips); therefore, it might be difficult to generalize our proposed deep learning models. Further studies validating our model using MR images from multiple institutions are needed. Third, more study will need to improve the generalizability of our model for clinical implications. Even though the prediction performance of an MRI-clinical information fused model is best in our experiments, it is difficult to expect the same environment as the experimental conditions as both refined clinical information and two different MR images for model inference in a clinical setting. Considering that clinical information is readily available regardless of medical institution, the clinical information only model may have a lower performance than the fused model, but the generalizability may be higher.

Interestingly, when comparing the cases about models trained with MR images and only clinical information, the sensitivity was higher in the clinical information-only model and the specificity was higher in all the models trained with MR images (Table 2). The clinical information-only model is supposed to predict pCR relatively consistently by learning standardized clinical information such as subtype or receptor status. However, in the model that was trained with MR images, it seems that the sensitivity decreases due to the increase of false negatives (interpreted as non-pCR but actually pCR) because the chemotherapy-related changes such as fibrosis or inflammation may lead to incorrect predictions. In addition, the definition of pCR in our study was pT0/is N0, which means residual ductal carcinoma in situ (DCIS) was included as pCR. Differentiation between DCIS and invasive carcinoma is almost impossible. Therefore, residual DCIS could partly contribute to decreased sensitivity. Results might be different if we use different definition of pCR. This issue might be partly solved if functional images such as a diffusion MR images are added to the training dataset^30^. Further study is needed.

We selected T2W and T1W subtraction images as MR sequences for this study because they are most commonly used by radiologists to diagnose breast cancer and reflect tumor biology. If we additionally apply contrast-enhanced images from multiple post-contrast time points that were not used in this study, we can use kinetic information as leverage for predicting pCR^31^. Moreover, addition of diffusion-weighted imaging (DWI) can further improve the performance of the model. DWI is used to measure apparent diffusion coefficients (ADCs) on the breast tissue. ADC, a measure of the diffusivity of water within the tissue, is sensitive to intratumoral changes induced by chemotherapy. According to few clinical studies conducted on patients with breast cancer, the use of DWI is valuable in predicting pCR to NAC^10,13,32^. As we wrote before, in our study, sensitivity of MR image-based model was lower than clinical variable-only model. We anticipate that this could be improved when functional images such as a DWI are added to the training dataset for further study although strict standardization for obtaining consistent ADC value would be necessary.

After further studies with larger numbers of patients and various types of MR images, the proposed system might be used as an assisting tool for a pCR prediction on volumetric MR images and clinical information. Our model could be embedded software for MRI scanners for predicting pCR like MRI CAD (computer aided detection) software^33^. From the viewpoint of studies many drug therapies in the NAC setting, assessment of pCR is the standard primary end point, because pCR is the most important clinical characteristic to indicate the risk of recurrence and long-term survival^5,6^. Through the pCR prediction result of the deep learning model, the clinician may change treatment plans for breast cancer during NAC to improve survival of patients.

In summary, we conducted a study on the performance improvement of predictive model of pCR to NAC using both clinical information and pretreatment breast MR images by a multimodal fusion approach of the deep learning method. Based on the results presented in this study, we believe that a multimodal fusion approach for pCR prediction can be adopted in the development of a computer-aided diagnosis system that uses breast MR images. With further technological development, a pCR prediction model based on pretreatment MR images may be developed, and the system could aid in clinical decision-making for the NAC in patients with breast cancer.

## Methods

### Study cohort

This retrospective study was conducted in accordance with the Declaration of Helsinki and was approved by the Institutional Review Board of ethics committee from Samsung medical center (Seoul, Korea). The requirement for informed consent waived (IRB No. 2019-04-021). We reviewed patients with breast cancer who were treated with NAC at SMC between January 2010 and August 2018. The inclusion criteria for this study were as follows: (1) NAC with no prior therapy; (2) unilateral biopsy-proven primary breast cancer; (3) surgery after completion of NAC at our institution; and (4) breast MR images within 1 month prior to initiation of therapy at our institution. Patients who already had synchronous and metachronous double primary cancer or bilateral breast cancer were excluded. Finally, we enrolled a total of 536 patients with breast cancer who were treated with NAC in this study. The mean age of the patients was 45.2 years (range, 22–75 years; standard deviation, ± 9.9).

The NAC regimen was classified into four categories: adriamycin with cyclophosphamide (AC), AC followed by docetaxel (AC-T), AC-T plus platinum, and AC-T with trastuzumab. We treated breast cancer patients with standard NAC regimen as below: (1) adriamycin (60 mg/m^2^) with cyclophosphamide (600 mg/m^2^) iv every 3 weeks for six cycles; (2) adriamycin (60 mg/m^2^) with cyclophosphamide (600 mg/m^2^) iv every 3 weeks for four cycles followed by taxane iv every 3 weeks for another four cycles; or 3) docetaxel (75 mg/m^2^), and carboplatin (area under the curve 5.5). In HER2-positive breast cancer, adriamycin (60 mg/m^2^) with cyclophosphamide (600 mg/m^2^) iv every 3 weeks for four cycles followed by taxane with trastuzumab (8 mg/kg at cycle 1, 6 mg/kg at cycles 2–4) iv every 3 weeks for another four cycles. After surgery, patients received adjuvant trastuzumab every 3 weeks for a year.

The mean interval between treatment initiation and surgery was 197.0 days (range, 96–614 days). Duration of examination depended on the schedule of NAC.

### MR image acquisition and preprocessing

The breast MR examination consisted of turbo spin-echo T1W and T2W sequences and 3D dynamic contrast-enhanced (DCE) sequence for each patient. T2W and contrast-enhanced T1W subtraction MR images were retrieved from the picture archiving communication system and loaded onto a workstation for further analysis. Subtraction images from contrast-enhanced images at 90 s after contrast injection to pre-enhanced images were selected in this study. The detailed description of the MR image acquisition procedure is as follows: All magnetic resonance imaging (MRI) scans were performed on either a 1.5-T or a 3.0-T scanner from Philips (1.5-T Achieva and 3.0-T Achieva, Philips Healthcare, Best, Netherlands) with a dedicated bilateral phased-array breast coil. The DCE-MRI scans on a 1.5-T scanner (n = 254) were acquired using the following parameters: TR/TE, 6.5/2.5; slice thickness, 1.5 mm; flip angle, 10°; matrix size, 376 × 374; and field of view, 32 × 32 cm. DCE-MRI was performed with axial imaging with one pre-contrast and six post-contrast dynamic series. Contrast-enhanced images were acquired at 0.5, 1.5, 2.5, 3.5, 4.5, and 5.5 min after contrast injection. The DCE-MRI scans on a 3.0 T scanner (n = 282) were acquired using the following parameters: TR/TE, 5.5/2.8; slice thickness, 3 mm; flip angle, 12°; matrix size, 500 × 237; and field of view, 30 × 30 cm. DCE-MRI was performed with axial imaging, with one pre-contrast and six post-contrast dynamic series. Contrast-enhanced images were acquired at 0.5, 1.5, 2.5, 3.5, 4.5, and 5.5 min after contrast injection. Image subtraction was performed after the dynamic series. To obtain the DCE T1W subtraction image as model input, images that were acquired before injection were subtracted from the images that were acquired at 1.5 min after contrast injection. For dynamic contrast enhancement, a 0.1 mmol/kg bolus of gadobutrol (Gadovist; Bayer HealthCare Pharmaceuticals, Berlin, Germany) was injected, followed by a 20 mL saline flush.

We applied MR image preprocessing as follows: (1) interpolating different voxel dimensions of MR images to isotropic spacing (1.2 × 1.2 × 1.2 mm^3^) using nearest neighbor interpolation^34^; (2) normalizing various intensity of MR images, DCE T1W and T2W, using histogram-matching algorithm^35^; and (3) converting normalized MR images from 12-bit to 8-bit grayscale (pixel values ranging from 0 to 255). We conducted all abovementioned preprocessing algorithms using the SimpleITK Python package (version 1.2.0)^36^.

For comparison of the performance between the models trained with uncropped versus cropped images, cropped images of 56 × 56 × 12 voxel sizes containing lesions were obtained from the original DCE T1W subtraction images. The location of lesions was manually drawn slice-by-slice on the DCE T1W subtraction images by a breast radiologist with 14 years’ experience of reading breast MR images. From the center point measured from the lesion boundary, 3D cropped images were obtained.

### Clinicopathologic characteristics of patients

Pretreatment core biopsies were used to determine the receptor status of the tumor. pCR was defined as no residual invasive tumor in the breast and ipsilateral axilla (pT0/is N0). Patients were divided into pCR or non-pCR based on pathological report after final surgery. We collected the following clinicopathologic characteristics: age, body mass index (BMI), menopausal status, histologic subtypes, T stage, N stage, estrogen receptor (ER), progesterone receptor (PR), human epidermal growth factor receptor 2 (HER2), Ki-67, cancer antigen (CA) 15-3, and NAC regimen. ER, PR score were defined according to the Allred score. HER2 status was evaluated by immunohistochemical (IHC) staining using antibody with grade 3 + being positive, 0 and 1 + being negative, and 2 + requiring further evaluation by silver in-situ hybridization. CA 15-3 levels before NAC were reviewed from the medical records. Clinical T and N stages were determined according to TNM staging system by the American Joint Committee on Cancer 7th edition^35^. For convenience, histologic subtypes of breast cancer were divided into two groups: invasive ductal carcinoma (IDC) and others.

For clinical information preprocessing, we normalized numeric features, such as age, BMI, ER score, PR score, T stage, N stage, and CA 15-3 level, to the range between 0 and 1 via min–max normalization. Additionally, we converted categorical features, such as HER2, Ki-67, histologic subtype, and NAC regimen, into one-hot vectors.

### Model architecture and training

**Figure 2:**
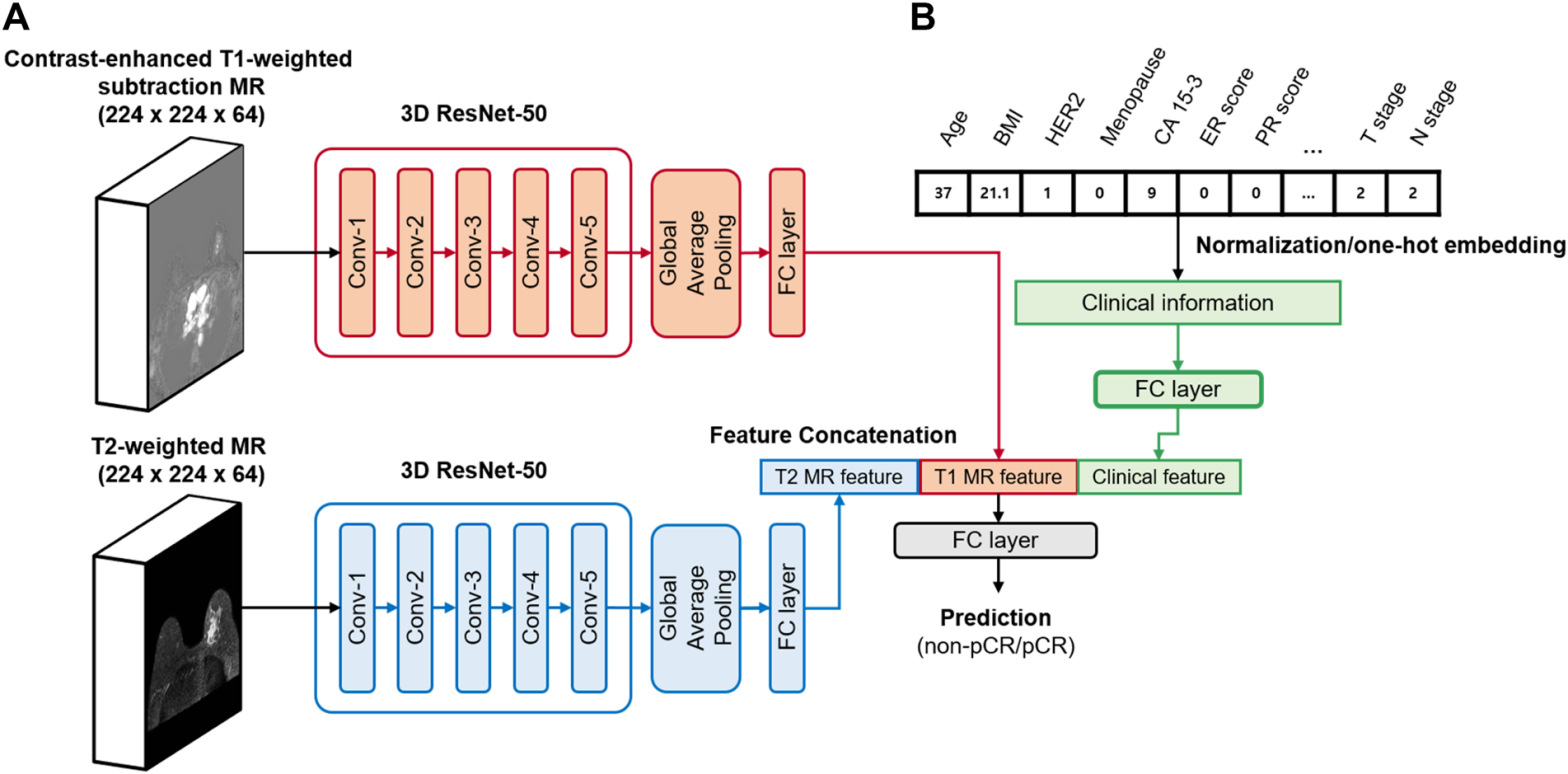
Deep learning architectures for the multimodal pCR prediction model. (**A**) The feature extractors for contrast-enhanced T1W subtraction MR images and T2W MR images were used in two 3D ResNet-50. The MR images for the input were subjected to isotropic transformation and cropped to a 3D form of 224 × 224 × 64. (**B**) FC layer was used for clinical information inputs. The outputs of each 3D ResNet-50 and FC layer for clinical information were concatenated. The final FC layer with sigmoid activation function was used in the prediction of pCR.

We applied a multimodal fusion method in the deep neural network to combine different types of data. We choose the 3D-ResNet architecture for extracting features from 3D-bilateral whole MR images. The 3D-ResNet has the skip-connection between layers in a network and tends to benefit from a reduced number of parameters^37^. It enables the trainable deeper structure and adds nonlinearity to the model for achieving adequate performance in classification. The proposed model consists of three parts: (1) 3D volumetric CNNs for feature extraction from MR images; (2) FC layers for clinical information and feature concatenation; and (3) final FC layer to allow prediction of pCR (Fig. 2). To achieve feature extraction from volumetric MR images, we modified the bottleneck 3D-ResNet-50 architecture designed by Hara et al.^37^ (Supplementary Table S2). In our study, contrast-enhanced T1W subtraction and T2W images with shapes of 224 × 224 × 64 voxels were separately entered into the 3D-ResNet-50. The outputs passed a FC layer consisting of six neurons. Finally, two 6D vectors from T1W subtraction and T2W images were extracted. Twelve clinical information passed through one FC layer consisting of six nodes with dropout rate of 0.1 (Fig. 2B). To fuse multimodal features, the two 6D vectors from T1W subtraction and T2W images and the 6D vector from clinical information were concatenated into an 18D vector. Finally, the concatenated output was passed through the final sigmoid layer, allowing a binary classification output. From the experiment, six dimensions were selected as feature vector size to achieve the best model performance.

We elaborate here about the training procedure of our model. Dropout with a probability of 0.1 was performed in the FC layers after feature extraction layer for MR images and clinical information to prevent overfitting during training. We divided 536 patients into 8:2 (429 for training, 107 for validation) using stratified random sampling, which ensures that subsets’ proportions are the same as the original set. We applied several data augmentation methods in the training phase as random cropping, rotating, and flipping to improve generalization of the model. The random cropping was parameterized to minimize the background in axial views in the crops (224 × 224 × 64 voxels). Axial MR images were randomly rotated at an angle ranging from − 10° to 10° and flipped left and right with probability of 50%. To address the concern of class imbalance issue, focal loss^38^ was used. We use the PyTorch^39^ (version 1.1.0) open-source deep learning framework as the main tool for generating the software implementation of the presented architecture. The training was conducted for 12 h on a DGX Station with a Tesla V100 GPU with the Rectified Adam optimizer^40^. The model also trained with a learning rate of 0.0354, weight decay of 0.00001, batch size of 8, and early stopping to avoid overtraining^41^.

### Statistical Analysis

We compared the difference between training and validation dataset with the t-test for mean of numeric variables and chi-square test or Fisher’s exact test for categorical variables (Python SciPy package). A *P* value < 0.05 indicated a statistically significant difference. We evaluated seven types of deep learning models trained on different combinations of clinical information, T1W subtraction images, and T2W images to analyze the effects of each dataset. The seven datasets to train each model consisted of the following: (1) dataset containing clinical information; (2) dataset containing T1W subtraction images; (3) dataset containing T2W images; (4) dataset containing cropped image for lesions in T1W subtraction images; (5) combined dataset containing T1W subtraction and T2W images; (6) combined dataset containing T1W subtraction images and clinical information; and (7) combined dataset containing T1W subtraction images, T2W images, and clinical information. The performance of each model was evaluated using AUC and confusion matrix. The significance of the AUC differences was tested using the method described by Hanley and McNeil^42^.

## Supplementary Information

Supplementary Information.

## References

[1] Curigliano G (2017). De-escalating and escalating treatments for early-stage breast cancer: the St. Gallen international expert consensus conference primary therapy of early breast cancer 2017. Ann. Oncol..

[2] Kaufmann M (2006). Recommendations from an international expert panel on the use of neoadjuvant (primary) systemic treatment of operable breast cancer: an update. J. Clin. Oncol..

[3] Wang-Lopez Q (2015). Can pathologic complete response (pCR) be used as a surrogate marker of survival after neoadjuvant therapy for breast cancer?. Crit. Rev. Oncol. Hematol..

[4] Mougalian SS (2015). Use of neoadjuvant chemotherapy for patients with stage I to III breast cancer in the United States. Cancer.

[5] Asselain B (2018). Long-term outcomes for neoadjuvant versus adjuvant chemotherapy in early breast cancer: meta-analysis of individual patient data from ten randomised trials. Lancet Oncol..

[6] Rastogi P (2008). Preoperative chemotherapy: updates of National Surgical Adjuvant Breast and Bowel Project Protocols B-18 and B-27. J. Clin. Oncol..

[7] Tewari M, Krishnamurthy A, Shukla HS (2008). Predictive markers of response to neoadjuvant chemotherapy in breast cancer. Surg. Oncol..

[8] Rouzier R (2005). Nomograms to predict pathologic complete response and metastasis-free survival after preoperative chemotherapy for breast cancer. J. Clin. Oncol..

[9] Fernández-Sánchez M (2006). Clinical and pathological predictors of the response to neoadjuvant anthracycline chemotherapy in locally advanced breast cancer. Med. Oncol..

[10] Park SH (2010). Diffusion-weighted MR imaging: pretreatment prediction of response to neoadjuvant chemotherapy in patients with breast cancer. Radiology.

[11] Braman N (2019). Association of peritumoral radiomics with tumor biology and pathologic response to preoperative targeted therapy for HER2 (ERBB2) positive breast cancer. JAMA Netw. Open.

[12] Cain EH (2019). Multivariate machine learning models for prediction of pathologic response to neoadjuvant therapy in breast cancer using MRI features: a study using an independent validation set. Breast Cancer Res. Treat..

[13] Liu Z (2019). Radiomics of multiparametric MRI for pretreatment prediction of pathologic complete response to neoadjuvant chemotherapy in breast cancer: a multicenter study. Clin. Cancer Res..

[14] LeCun Y, Bengio Y, Hinton G (2015). Deep learning. Nature.

[15] Xi IL (2020). Deep Learning to distinguish benign from malignant renal lesions based on routine MR imaging. Clin. Cancer Res..

[16] Titano JJ (2018). Automated deep-neural-network surveillance of cranial images for acute neurologic events. Nat. Med..

[17] Esteva A (2017). Dermatologist-level classification of skin cancer with deep neural networks. Nature.

[18] Peng H (2019). Prognostic value of deep learning PET/CT-based radiomics: potential role for future individual induction chemotherapy in advanced nasopharyngeal carcinoma. Clin. Cancer Res..

[19] El Adoui, M., Drisis, S. & Benjelloun, M. Predict Breast Tumor Response to Chemotherapy Using a 3D Deep Learning Architecture Applied to DCE-MRI Data. in *Bioinformatics and Biomedical Engineering. IWBBIO 2019. Lecture Notes in Computer Science* vol. 2 33–40 (Springer International Publishing, 2019).

[20] Ravichandran, K., Braman, N., Janowczyk, A. & Madabhushi, A. A deep learning classifier for prediction of pathological complete response to neoadjuvant chemotherapy from baseline breast DCE-MRI. in *Medical Imaging 2018: Computer-Aided Diagnosis* (eds. Mori, K. & Petrick, N.) 11 (SPIE, 2018). doi:10.1117/12.2294056.

[21] Ha R (2019). Prior to initiation of chemotherapy, can we predict breast tumor response? Deep learning convolutional neural networks approach using a breast MRI tumor dataset. J. Digit. Imaging.

[22] Le MH (2017). Automated diagnosis of prostate cancer in multi-parametric MRI based on multimodal convolutional neural networks. Phys. Med. Biol..

[23] Nie K (2016). Rectal cancer: assessment of neoadjuvant chemoradiation outcome based on radiomics of multiparametric MRI. Clin. Cancer Res..

[24] Nie D (2019). Multi-channel 3D deep feature learning for survival time prediction of brain tumor patients using multi-modal neuroimages. Sci. Rep..

[25] Gillies RJ, Kinahan PE, Hricak H (2016). Radiomics: images are more than pictures, they are data. Radiology.

[26] Cho, J., Lee, K., Shin, E., Choy, G. & Do, S. How much data is needed to train a medical image deep learning system to achieve necessary high accuracy? (2015).

[27] Topol EJ (2019). High-performance medicine: the convergence of human and artificial intelligence. Nat. Med..

[28] Fukui, H., Hirakawa, T., Yamashita, T. & Fujiyoshi, H. Attention Branch Network: Learning of Attention Mechanism for Visual Explanation (2018).

[29] Selvaraju, R. R. *et al.* Grad-CAM: Visual Explanations from Deep Networks via Gradient-Based Localization. in *Proceedings of the IEEE International Conference on Computer Vision* vols 2017-Octob 618–626 (2017).

[30] ElDaly MM, Moustafa AFI, Abdel-Meguid SMS, Shokry AM, Abd El Wahab N (2018). Can MRI diffusion-weighted imaging identify postoperative residual/recurrent soft-tissue sarcomas?. Indian J. Radiol. Imaging.

[31] Whitney HM (2019). Additive benefit of radiomics over size alone in the distinction between benign lesions and luminal a cancers on a large clinical breast MRI dataset. Acad. Radiol..

[32] Tahmassebi A (2019). Impact of machine learning with multiparametric magnetic resonance imaging of the breast for early prediction of response to neoadjuvant chemotherapy and survival outcomes in breast cancer patients. Invest. Radiol..

[33] Dorrius MD, Jansen-van der Weide MC, van Ooijen PMA, Pijnappel RM, Oudkerk M (2011). Computer-aided detection in breast MRI: a systematic review and meta-analysis. Eur. Radiol..

[34] Manjón JV (2010). Non-local MRI upsampling. Med. Image Anal..

[35] Nyúl LG, Udupa JK, Zhang X (2000). New variants of a method of MRI scale standardization. IEEE Trans. Med. Imaging.

[36] Yaniv Z, Lowekamp BC, Johnson HJ, Beare R (2018). SimpleITK image-analysis notebooks: a collaborative environment for education and reproducible research. J. Digit. Imaging.

[37] Hara, K., Kataoka, H. & Satoh, Y. Can Spatiotemporal 3D CNNs Retrace the History of 2D CNNs and ImageNet? in *2018 IEEE/CVF Conference on Computer Vision and Pattern Recognition* 6546–6555 (IEEE, 2018). doi:10.1109/CVPR.2018.00685.

[38] Lin, T. Y., Goyal, P., Girshick, R., He, K. & Dollar, P. Focal Loss for Dense Object Detection. in *Proceedings of the IEEE International Conference on Computer Vision* vols 2017-Octob 2999–3007 (2017).

[39] Paszke, A. *et al.* PyTorch: An Imperative Style, High-Performance Deep Learning Library (2019).

[40] Liu, L. *et al.* On the Variance of the Adaptive Learning Rate and Beyond (2019).

[41] Lin, J., Camoriano, R. & Rosasco, L. Generalization Properties and Implicit Regularization for Multiple Passes SGM (2016).

[42] Hanley JA, McNeil BJ (1983). A method of comparing the areas under receiver operating characteristic curves derived from the same cases. Radiology.

