# Supplementary material for: Multimodal deep learning models for the prediction of pathologic response to neoadjuvant chemotherapy in breast cancer
Source: Sci Rep. 2021 Sep 22;11:18800. doi: 10.1038/s41598-021-98408-8 (PMC8458289; doi:10.1038/s41598-021-98408-8)
Supplement: Supplementary file 1 — Supplementary Information. [file 41598_2021_98408_MOESM1_ESM.docx]

# SUPPLEMENTARY INFORMATION

**Supplementary Table S1**. Detailed network architectures of ResNet-50 with 3D-CNN. F is the number of feature channels, and N is the number of blocks in each layer.

| Model | Conv1 | Conv2 | | Conv3 | | Conv4 | | Conv5 | |  |
| --- | --- | --- | --- | --- | --- | --- | --- | --- | --- | --- |
|  |  | F | N | F | N | F | N | F | N |  |
| ResNet-50 with bottleneck block | Conv, 7ⅹ7ⅹ7, 64,  temporal stride 1,  spatial stride 2 | 64 | 3 | 128 | {4,4,  8,24} | 256 | {6,23,  36,36} | 512 | 3 | Global average pool,  6D fully connected,  sigmoid |

*Note*. Adapted from Hara et al. Can Spatiotemporal 3D CNNs Retrace the History of 2D CNNs and ImageNet? *2018 IEEE/CVF Conf Comput Vis Pattern Recognit*. IEEE; 2018. page 6546–55.

**Supplementary Table S2.** Demographic comparison between the training and validation cohorts

| **Characteristics** | **Training set**  **(N = 429)** | **Validation set**  **(N = 107)** | ***P-*value** |
| --- | --- | --- | --- |
| Non-pCR (n, %)  pCR (n, %) | 329 (76.69)  100 (23.31) | 74 (69.16)  33 (30.84) | 0.107 |
| Age (years, mean ± standard deviation) | 45.23 ± 10.21 | 45.18 ± 8.58 | 0.964 |
| BMI (kg/m^2^, mean ± standard deviation) | 23.14 ± 3.29 | 23.17 ± 3.27 | 0.951 |
| Menopausal status (n, %)  Premenopausal  Postmenopausal | 295 (68.76)  134 (31.24) | 78 (72.90)  29 (27.10) | 0.406 |
| ER (n, %)  Positive  Negative | 188 (43.82)  241 (56.18) | 49 (45.79)  58 (54.21) | 0.713 |
| PR (n, %)  Positive  Negative | 155 (36.13)  274 (63.87) | 39 (36.45)  68 (63.55) | 0.951 |
| HER2 status (n, %)  Positive  Negative | 143 (33.33)  286 (66.67) | 34 (31.78)  73 (68.22) | 0.759 |
| Clinical T-stage at diagnosis (n, %)  cT1  cT2  cT3  cT4 | 17 (3.96)  224 (52.21)  149 (34.73)  39 (9.09) | 9 (8.41)  64 (59.81)  29 (27.10)  5 (4.67) | 0.047 |
| Clinical N-stage at diagnosis (n, %)  cN0  cN1  cN2  cN3 | 31 (7.23)  84 (19.58)  184 (42.89)  130 (30.30) | 11 (10.28)  24 (22.43)  46 (42.99)  26 (24.30) | 0.489 |
| CA 15-3 level (U/mL, mean ± standard deviation) | 17.42 ± 23.97 | 17.92 ± 21.88 | 0.844 |
| Ki-67 (n, %)  +1  +2  +3  +4 | 80 (18.65)  142 (33.10)  110 (25.64)  97 (22.61) | 22 (20.56)  37 (34.58)  18 (16.82)  30 (28.04) | 0.254 |
| Pathologic diagnosis (n, %)  IDC  Others | 380 (88.58)  49 (11.42) | 101 (94.39)  6 (5.61) | 0.076 |
| NAC regimen (n, %)  AC-T  ACTH  AC-T & Platinum  AC | 292 (68.07)  104 (24.24)  13 (3.03)  20 (4.66) | 75 (70.09)  27 (25.23)  2 (1.87)  3 (2.80) | 0.756 |

pCR, pathologic complete response; BMI, body mass index; ER, estrogen receptor; PR, progesterone receptor; HER2, human epidermal growth factor receptor 2; CA 15-3, cancer antigen 15-3; NAC, neoadjuvant chemotherapy


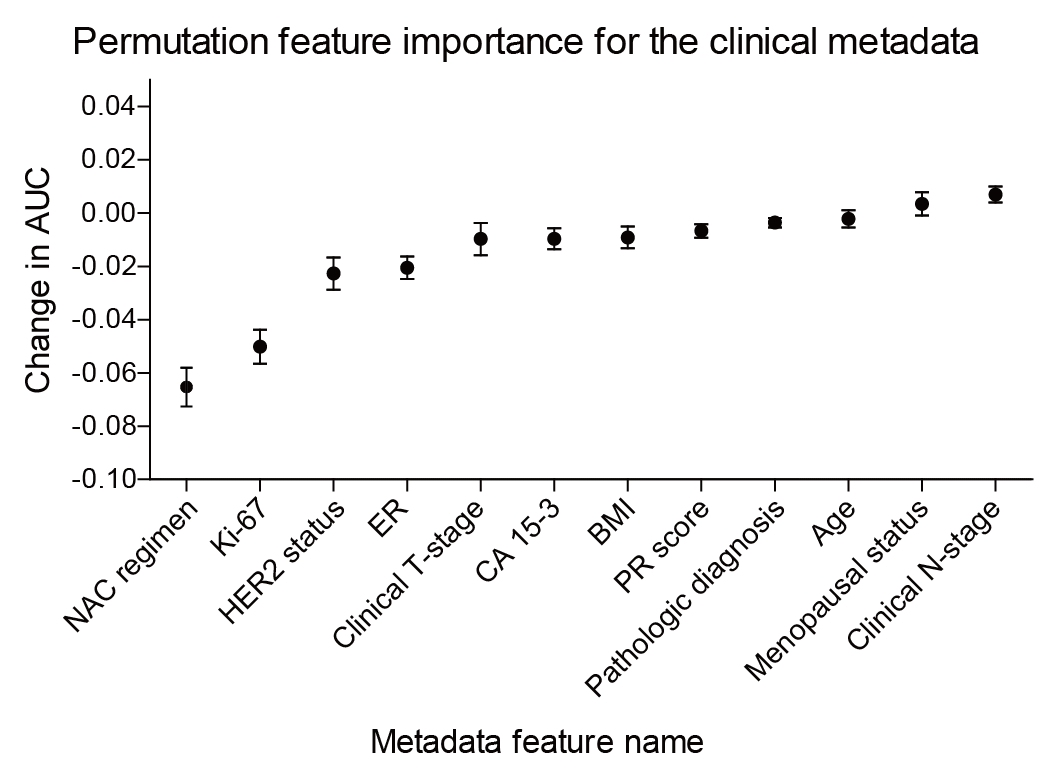


Supplementary Figure S1. Permutation feature importance in the clinical information


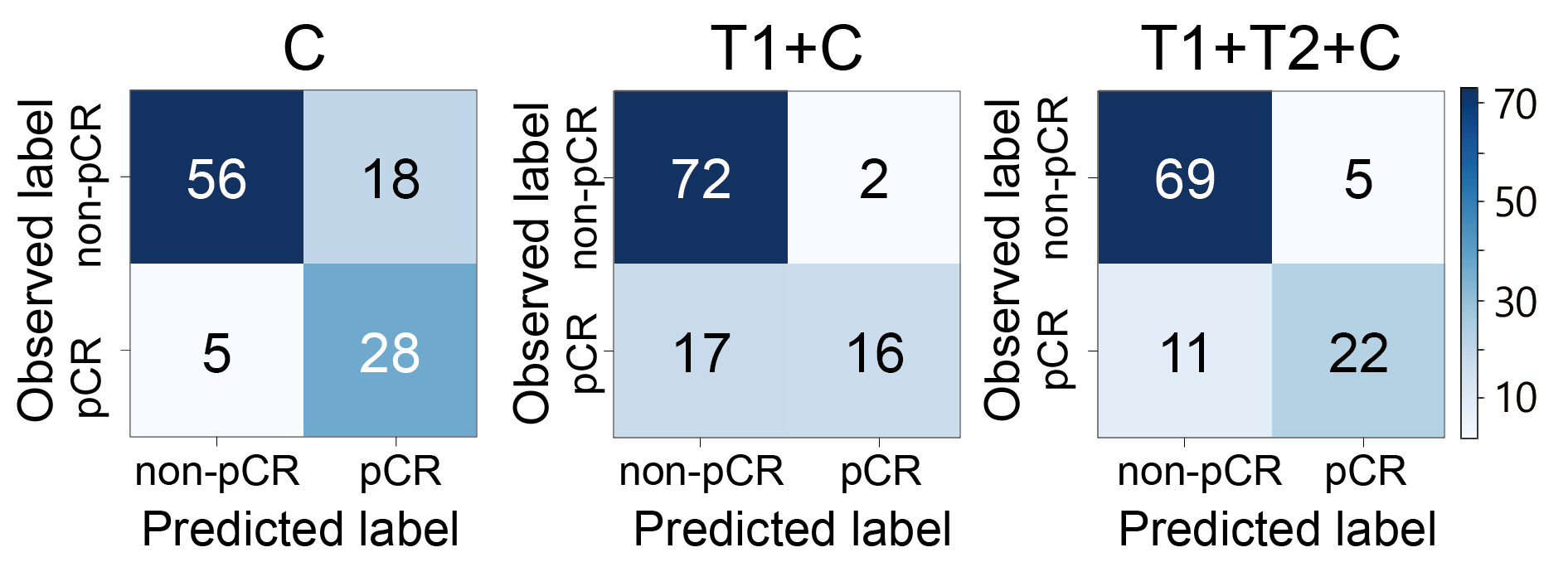


**Supplementary Figure S2.** Confusion matrices of different classifiers. C, clinical information; T1+C, T1W subtraction images and clinical information; T1+T2+C, T1W subtraction images, T2W images, and clinical information
